# Catheter Free Day of Surgery Discharge vs Overnight Observation Following Artificial Urinary Sphincter Placement

**DOI:** 10.7759/cureus.36898

**Published:** 2023-03-30

**Authors:** John M Myrga, Robin Vasan, David T Miller, Christopher J Staniorski, Cory Taylor, Paul Rusilko

**Affiliations:** 1 Urology, University of Pittsburgh Medical Center, Pittsburgh, USA; 2 Urology, Univeristy of Pittsburgh Medical Center, Pittsburgh, USA

**Keywords:** clinician-measured outcomes, same day discharge, artificial urinary sphincter, urology, #male urethra

## Abstract

Introduction

To confirm the safety and examine outcomes of a day of surgery discharge following artificial urinary sphincter implantation in a population discharged without a catheter.

Methods

We retrospectively identified 110 patients, 31 of whom were discharged on the day of surgery, from a single surgeon following artificial urinary sphincter implantation. After institutional board review approval, patient charts were reviewed capturing demographics as well as three, thirty, and ninety-day outcomes. Further outcomes specific to urinary retention were obtained.

Results

Patients who were discharged the same day were older (71 vs. 68), had shorter operative times (92 minutes vs 109 minutes), and were less likely to have been smokers (6% vs 31%). There were no differences in the proportion of patients who underwent prior radiation or prior implant surgery. There was no significant difference in the number of patients who had emergency department visits, urinary retention, office calls, office visits, or unplanned office visits at all time points following surgery. There was no significant difference in overall urinary retention (15% vs 5%), retention presenting after the initial surgical event (6% vs 5%), or need for a suprapubic tube (0% vs 5%).

Conclusions

Day of surgery discharge is a safe discharge strategy for patients who have undergone artificial urinary sphincter placement. Furthermore, catheter-free days of discharge surgery did not have a significantly greater risk of urinary retention, office calls, emergency department (ED) visits, or office visits compared to our overnight observation population. This approach should be considered for all patients undergoing artificial urinary sphincter (AUS) implantation.

## Introduction

Artificial Urinary Sphincter (AUS) implantation is a common surgical treatment for men with urinary incontinence. The current post-operative standard of care requires an overnight stay with intravenous antibiotics and catheter removal the day following surgery. The COVID-19 epidemic and subsequent patient bed and staff shortages have necessitated new strategies to reduce the utilization of hospital resources. These interventions include promoting day-of-surgery discharges and avoidance of hospital admissions. Prior studies show that penile implants can be safely discharged home from a surgical and anesthesia standpoint on the day of surgery with high patient satisfaction [1]. Additional studies have shown a low risk of immediate complications and the need for narcotics following AUS implantation [2]. Another recent study shows day of surgery discharge following AUS implantation with a urinary catheter in place is a safe management strategy from both a surgical and anesthesia standpoint [3]. 

The concept of a day of surgery discharge in an AUS population is evolving and there continue to be variations in surgical approach. The only studied strategy has utilized a urinary catheter on discharge, but many patients are uncomfortable with the management or presence of a urinary catheter [4]. Avoiding urinary catheters following surgery has also been shown to reduce the risk of urinary infection [5,6]. Patients who cannot or feel uncomfortable removing their catheter following surgery may require an additional office visit. For patients who live far from their implantation site or a local urologist, this may require excess travel, sway providers into keeping a patient for an overnight stay or prevent patients from having an implant altogether. For these patients, avoiding a catheter may be an important aspect of their discharge strategy. Our study examined outcomes associated with same-day catheter-free discharge after AUS implantation to confirm safety and expand discharge strategies. Data in this paper was previously presented as an abstract at the 2022 American College of Surgeons Clinical Congress in San Diego, CA in October of 2022.

## Materials and methods

With institutional approval, all AUS placements since 2015 from a single surgeon were retrospectively identified. In 2020, we transitioned to a discharge strategy where a catheter was placed intra-operatively and removed at the end of surgery or in the post-anesthesia care unit prior to discharge. All AUS patients at our institution, regardless of discharge strategy, underwent general anesthesia. Post-void residuals were obtained to ensure adequate emptying. Data regarding patient demographics, emergency department (ED) visits, postoperative urinary retention, and office interactions were abstracted from the medical record. We examined outcomes at the three, thirty, and ninety-day time points. 

Prior to 2020, our protocol followed standard recommendations for AUS placement. Patients were given a dose of intravenous antibiotics before the procedure, a 12 Fr silicone catheter was placed intra-operatively and patients were kept overnight. Intravenous antibiotics were continued until discharge. The morning following surgery, the catheter was removed and patients were discharged following confirmation of a low post-void residual. Patients were prescribed a short course of opioids and antibiotics. Patients were seen in the office at the two-week and then six-week mark.

Our day of surgery discharges had the following general principles. As in the standard fashion, patients were given a dose of intravenous antibiotics in the operating room and a 12 Fr silicone catheter was placed. The catheter was removed in the operating room or in the post-anesthesia care unit and a patient was required to pass a voiding trial with a post-void residual less than voided volume prior to discharge. Any patient who was unable to empty their bladder post-operatively had a 12 Fr silicone catheter placed and a follow-up appointment in 72 hours for a voiding trial. Perioperative opioid-sparing protocols were initiated wherever possible and patients were not routinely discharged with narcotics post-operatively. Patients were prescribed a short course of antibiotics. Pain management consisted of over-the-counter acetaminophen and ibuprofen. Patients were scheduled to follow up in four to six weeks for device activation. 

Post-operative urinary retention was defined as any need for catheter placement in the postoperative setting. Patients were instructed to call the office with any concerns and given strict return precautions if they felt they had difficulty emptying their bladder. In our non-day-of-discharge population, patients who were unable to void typically underwent repeat short-term urethral catheter placement or placement of a suprapubic tube per physician preference. 

For our statistical analysis, categorical variables were compared using Chi-square or Fisher's exact test as appropriate. For continuous variables, normality was assessed using the Kolmogorov-Smirnov test. Normally distributed variables were analyzed using Student's t-test and outcomes recorded as mean with standard deviation. Non-normally distributed data were analyzed using a Mann-Whitney Test with outcomes recorded as median with interquartile range. All statistics were run using SPSS (IBM Corp. Released 2021. IBM SPSS Statistics for Windows, Version 28.0. Armonk, NY: IBM Corp).

## Results

A hundred and ten patients were included, 31 of whom were discharged on the day of surgery. Patients who were discharged the same day were older (71 vs. 68, p=0.04), had shorter operative times (92 minutes vs 109 minutes, p<0.01), and were less likely to have been smokers (6% vs 31%, p<0.01). There were also no differences in the proportion of patients who underwent prior radiation or prior implant surgery. When examining outcomes, there was no significant difference in the number of patients who had emergency department visits, urinary retention, office calls, office visits, or unplanned visits at all time points following surgery (Table 1). Of note, we did not have any postoperative hematomas throughout both arms of our study. Looking at outcomes specific to catheter removal day of surgery, there was no significant difference in overall urinary retention (15% vs 5%, p=0.22), retention seen after the initial surgical event (6% vs 5%, p=0.99), or need for suprapubic tube (0% vs 5%, p=0.58).

**Table 1:** Three, thirty, and ninety day outcomes of patients undergoing artificial urinary sphincter placement.

Outcome	Day of Surgery Discharge (n=31) (Number, %)	Post Operative Admission (n=79) (Number, %)	P-Value
Post Op Retention Requiring Catheter	4 (15)	4 (5)	0.22
Post Op Retention Requiring Presentation to ED or Office	2 (6)	4 (5)	0.99
Post Op Retention Requiring Suprapubic Tube	0 (0)	4 (5)	0.58
Three Day Outcomes			
Emergency Department Visit	1 (3)	4 (5)	0.99
Any Call	5 (16)	12 (15)	0.61
Unplanned Office Visit	2 (6)	3 (4)	0.62
Readmission	0 (0)	0 (0)	-
Complication	0 (0)	3 (4)	0.56
Thirty Day Outcomes			
Emergency Department Visit	2 (6)	8 (10)	0.72
Any Call	11 (35)	27 (34)	0.99
Unplanned Office Visit	5 (16)	12 (15)	0.99
Readmission	1 (3)	5 (6)	0.99
Complication	1 (3)	3 (4)	0.99
Ninety Day Outcomes			
Emergency Department Visit	6 (19)	11 (14)	0.57
Any Call	17 (55)	33 (42)	0.29
Unplanned Office Visit	12 (39)	20 (25)	0.17
Readmission	1 (3)	10 (13)	0.18
Complication	2 (6)	7 (9)	0.99

## Discussion

We have corroborated evidence that day-of-surgery discharge after AUS implantation is a safe strategy. Furthermore, we have shown the feasibility of day-of-surgery discharge without a urinary catheter. In our study, we examined 31 patients who were discharged the same day following surgery without a catheter and compared them to 79 patients discharged following standard protocol. Importantly, this population consisted of patients who underwent both straightforward and complex implants. We did not exclude patients based on the complexity of their AUS surgery. We showed there was no increased risk of presentation to the emergency department, calls or visits to the office, or need for readmission to the hospital. Furthermore, looking at outcomes that would be specific to catheter removal, there was not a significant difference in the rate of postoperative urinary retention. Day-of-surgery discharges are safe and feasible after AUS implantation and urinary catheter placement on discharge are not routinely necessary.

A recent study has shown that day-of-surgery discharge after AUS is safe for patients; however, in these populations, catheters have been left in place on discharge [3]. While this is often viewed as a minor inconvenience, catheters increase the risk of infection, can be accidentally removed, and can fail to work as intended. For patients who travel far distances or are uncomfortable removing their own catheters, this also poses a problem regarding their follow-up care. Catheter removal in the immediate post-operative setting has often been considered risky; however, most large studies have shown only a 3%-5% risk of post-operative retention when catheters are removed the morning after surgery [3,7-9]. It is then reasonable to question whether a catheter placement is needed following the surgery.

Our study shows that there is no significant difference at any point in the need for a catheter between patients who were discharged day-of-surgery without a catheter as well as those who stayed overnight (15% vs 5%). In our study population, the absolute number of patients who required a catheter at any point following initial removal was only 4/31 patients. Two of these patients had catheters placed immediately in the post-anesthesia care unit and the other two patients presented the next day with incomplete voiding symptoms. 85% of patients in our study avoided a catheter overall and only 7% of patients ended up returning to the emergency department or the office for a catheter following the study. Of note, all patients who had retention requiring a catheter had the catheter removed within the first week following surgery. 

We did note some differences in baseline demographic characteristics between both populations. Same-day discharge patients were noted to be older and have higher rates of smoking than those discharged by the standard protocol. As we did not specifically select or screen patients for our day or surgery discharge, these differences are likely related to trends in patients electing surgery. Operative time was also noted to be shorter in the day-of-discharge population. As there is no significant time difference in the need for catheter placement, shorter operative times are likely related to increased provider operative efficiency [10]. Due to our small sample size, analyses examining which factors may lead to success in same-day catheter-free discharges could not be obtained. Additional large studies are needed to determine which factors may predict which patients can be discharged on the day of surgery following catheter removal. In addition, all of our outcomes are abstracted from patient charts. Future studies using patient-centered surveys may be useful to further elucidate a patient's perspective on their surgical experience. 

## Conclusions

Catheter-free day of discharge surgery did not have a significantly greater risk of urinary retention, office calls, emergency department (ED) visits, or office visits compared to our standard discharge population. This approach can also safely be utilized in both straightforward as well as complex implantations. For appropriate patients, day of surgery catheter-free discharge is a safe management strategy.
